# Cross-Sectional Study of Osteopathic General Surgeons in University-Based General Surgery Departments

**DOI:** 10.7759/cureus.24791

**Published:** 2022-05-06

**Authors:** Mustafa T Khan, Ronit Patnaik, Cassidy Wheeler, Mira Ibrahim, Haley Wolf, Kyle C Baumgardner, Rehana S Lovely

**Affiliations:** 1 General Surgery, University of Texas (UT) Health San Antonio, San Antonio, USA; 2 Texas College of Osteopathic Medicine, University of North Texas Health Science Center, Fort Worth, USA; 3 Long School of Medicine, University of Texas (UT) Health San Antonio, San Antonio, USA; 4 Center for Anatomical Sciences, University of North Texas Health Science Center, Fort Worth, USA

**Keywords:** academic productivity, publication, general surgery, osteopathic surgery, osteopathic medicine, academic surgery, surgical education, medical education

## Abstract

Introduction: Discrepancy between osteopathic (DO) and allopathic (MD) graduates in general surgery spans across all levels of training. In this cross-sectional study, we characterized DO surgeons who serve as faculty at university-based general surgery departments.

Methods: Overall, 106 university-based surgery departments were reviewed. DO and MD surgeons from the same institutions were identified, and demographic data were tabulated. MD surgeons were the control group. Univariate analysis and multivariate regression models were used to compare total publications, h-index, and citations.

Results: A total of 70 DO surgeons from 34 institutions were identified: 53 assistant professors, 16 associate professors, and one full professor. Of the DO surgeons, 35.7% completed residency at a university-based program, and 92.9% completed a fellowship, with surgical critical care and trauma being the most common. They were compared to 1,307 MD surgeons from the same institutions. Univariate analysis showed that MD faculty graduated medical school earlier (mean years (standard deviation (SD)): 14.8 (6.0) versus 23.3 (10.6); p<0.0001), had more total publications (median (interquartile range (IQR)): 5 (2.0-18.3) versus 35 (15.0-79.0); p<0.0001), had higher number of citations (median (IQR): 61.0 (14.0-265.0) versus 655.0 (155.0-2267.0); p<0.001), and had a higher h-index (median (IQR): 3 (1.0-8.0) versus 12 (6.0-24.0); p<0.001). Negative binomial regression models accounting for years since graduation, gender, and degree were performed. At the assistant professor rank, MD surgeons had more total publications (exponential coefficient (CI): 2.24 (1.67-3.02); p<0.001), more citations (3.10 (2.20-4.11); p<0.001), and a higher h-index (1.93 (1.36-2.73); p<0.001). Similar trends were noted at the associate professor level with MD surgeons having more total publication (1.67 (1.00-2.79); p=0.049), more citations (3.63 (2.13-6.18); p<0.001), and higher h-index (1.93 (1.10-3.39); p=0.022).

Conclusions: To address this discrepancy between DO and MD faculty surgeons, action must begin at the medical school and continue through residency. DO trainees need better access to mentorship and research support to foster an academic career.

## Introduction

The rise in osteopathic medical schools in the USA has resulted in an increase in the number of osteopathic (DO) applicants to residency programs across all specialties. In the 2022 residency match, US DO seniors filled 13.1% of the available general surgery categorical positions, a significant increase from 3.1% in 2012 [1,2].

Despite this increase in DO graduates entering general surgery residency training, osteopathic physicians remain underrepresented in general surgical practice, making up only 4.6% of all general surgeons in 2019 [3]. Multiple factors may be linked to this discrepancy. Osteopathic physicians may encounter subconscious bias during residency selection and training, lack of mentorship, a dearth of resources for research, and inadequate exposure to academic surgery programs compared to their allopathic counterparts [4-6]. For this reason, many osteopathic students interested in general surgery must go beyond their own institution to residency programs at other academic institutions to seek mentorship and research.

This added hurdle faced by osteopathic students may limit the number of students entering academic general surgery residency. Furthermore, this lack of representation in academic residency programs manifests as fewer osteopathic surgeons joining academic surgery departments as faculty. While the barriers for DO students to enter surgical residency have previously been studied, there is a lack of literature describing osteopathic surgeons who eventually pursue careers in academic general surgery as attending physicians [4,5]. To fill this gap in knowledge and to serve as a guide for future academic osteopathic surgeons, we performed a cross-sectional analysis of osteopathic faculty at university-based academic surgery departments and characterized their academic credentials.

## Materials and methods

All academic general surgery programs from a list compiled by Keane et al. were selected for this study [7]. This 2021 list was compiled by ranking the academic productivity of 106 university-based general surgery departments based on faculty lifetime h-index, faculty five-year h-index, total funding from the National Institute of Health (NIH), total funding from the Veterans Association (VA), and faculty involvement in journal editorial positions. Departmental websites were surveyed from April 2, 2022, to April 12, 2022. Only academic faculty with a DO degree were included in the analysis if they completed a general surgery residency in the USA, had an active clinical practice, and held an academic rank. A cohort of surgeons with an allopathic medical degree (MD) was also selected from the same departments to serve as the control group.

The following information was obtained from each surgery department’s website: faculty name, sex, academic rank, medical degree (MD versus DO), degree conferring medical school, year of medical school graduation, residency program attended, fellowship training program attended, and subspecialty. The American Medical Association’s FRIEDA^TM^ website was used to determine if the faculty member’s residency program was university-based, university-affiliated, or community-based [8].

Next, an individual search for every faculty was performed on the Scopus^TM^ database. Their names were cross-referenced with the name of the intuition at which they hold an academic rank. If an individual faculty had more than one search result, the results were combined. The degree of the number of citations, total publications, and h-index were tabulated.

Descriptive analysis was used to determine the proportion of sex (male versus female), academic rank (assistant professor, associate professor, and professor), type of residency training program (university-based versus university-affiliated or community-based), fellowship completion, and subspecialty. Since total publications, h-index, and the number of citations from the Scopus^TM^ bibliography were not normally distributed, we calculated the median and interquartile range (IQR) for both DO faculty and the MD faculty and compared them using Mood’s median test. The mean (standard deviation (SD)) years since medical school graduation was calculated and compared using the Mann-Whitney U test. The association between total publication, h-index, and the number of citations was further analyzed using multivariate negative binomial regression models to adjust for factors such as years since medical school graduation and gender of the faculty. In all our regression models, female gender and DO degree were considered the referent. All analyses were performed using SPSS statistical software version 28.0.1.0 (IBM Corp., Armonk, NY, USA). Statistical significance was set at 0.05.

Data used for this study was publicly available from university websites and stored without any identifying information. This study was determined by the University of Texas (UT) Health San Antonio IRB to be a nonhuman study as defined by the Department of Health and Human Service (DHHS) regulations at 45 CFR 46 and FDA regulations at 21 CFR 56. IRB review was not required.

## Results

After surveying 106 academic general surgery departments, a total of 70 academic DO surgeons spanning 34 institutions were identified (Table 1). Within this group, 53 were assistant professors, 16 were associate professors, and one was a full professor. Overall, 1,307 MD surgeons were also identified from the same institutions as the control group. In the MD group, 535 were assistant professors, 362 were associate professors, and 410 were full professors.

**Table 1:** Demographics of MD and DO surgeons

Variables	DO	MD
Total faculty	70	1,307
Male	40	869
Female	30	438
Academic programs	34	34
Assistant professor	53	535
Associate professor	16	362
Professor	1	410

**Figure 1:**
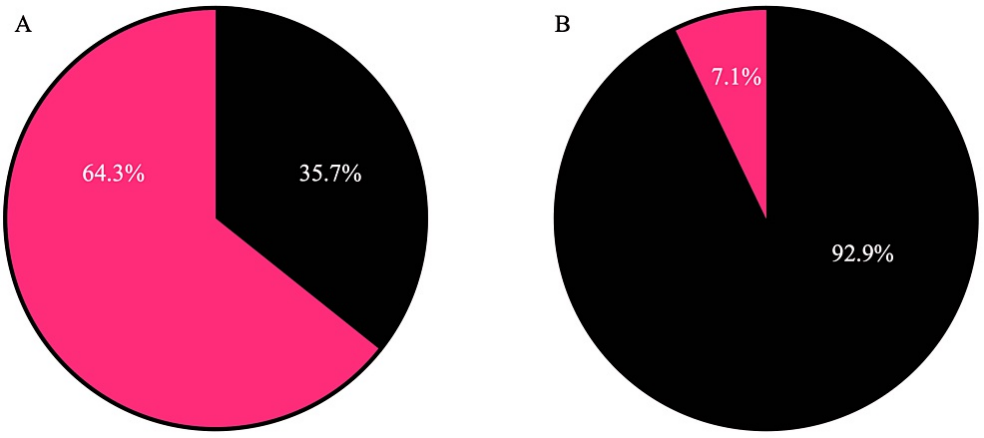
DO surgeon residency and fellowship training A: Percentage of DO faculty who completed their residency at university-based academic centers (black) versus community or university-affiliated center (pink). B: Percentage of DO faculty who completed a surgical fellowship (black) versus did not finish a surgical fellowship (pink).

In terms of academic qualifications, 25 of 70 DO general surgery faculty had completed their general surgery residency at university-based programs, while the rest (n=45) were at community or university-affiliated general surgery residency programs (Figure 1A). Of the 70 DO faculty, 65 completed an accredited surgical fellowship beyond their general surgery training (Figure 1B). In terms of subspecialty, most faculty were in surgical critical care and trauma surgery (n=30), followed by breast surgery (n=13), minimally invasive surgery (n=12), colorectal surgery (n=4), surgical oncology (n=3), and transplant surgery (n=3) (Table 2).

**Table 2:** Subspecialty breakdown of DO surgeons

Subspecialty	Number (%)
Surgical critical care and trauma surgery	30 (41.9%)
Breast surgery	13 (18.6%)
Minimally invasive surgery	12 (17.1%)
Colorectal surgery	4 (0.6%)
Surgical oncology	3 (0.4%)
Transplant surgery	3 (0.4%)
General surgery (non-fellowship trained)	5 (7.1%)

Univariate analysis showed that MD faculty had graduated medical school earlier than DO faculty (p<0.0001), had more total publications (p<0.001), had a higher number of citations (p<0.001), and a higher h-index (p<0.001) (Table 3).

**Table 3:** Univariate analysis between MD and DO Mood’s median test and Mann-Whitney U tests were performed. Significance was set at 0.05. IQR: interquartile range; SD: standard deviation

Academic metric	DO	MD	p-value
Total publication, median (IQR)	5 (2.0-18.3)	35.0 (15.0-79.0)	<0.001
Years since medical school graduation, mean (SD)	14.8 (6.0)	23.3 (10.6)	<0.001
Number of citations, median (IQR)	61.0 (14.0-265.0)	655.0 (155.0-2267.0)	<0.001
H-index, mean (SD)	3.0 (1.0-8.0)	12.0 (6.0-24.0)	<0.001

To account for potential confounding factors, negative binomial regression models accounting for years since medical school graduation, gender, and degree were performed (Table 4). Our results noted that at the assistant professor rank, MD faculty had more total publications (exponential coefficient (CI): 2.24 (1.67-3.02); p<0.001), more citations (3.10 (2.20-4.11); p<0.001), and a higher h-index (1.93 (1.36-2.73); p<0.001). Similar trends were noted at the associate professor level with MD faculty having more total publications (1.67 (1.00-2.79); p=0.049), more citations (3.63 (2.13-6.18); p<0.001), and higher h-index (1.93 (1.10-3.39); p=0.022). There was only one DO faculty member that was a full professor, so a comparison was not performed at the professor rank.

**Table 4:** Multivariate analysis of total publications, citations, and h-index Multivariate negative binomial regression was performed. Models included gender, years since medical school graduation, and degree. Referent conditions included female gender and DO degree.

Variables	Exponential coefficient (CI)	p-value
Assistant professor		
Total publications	2.24 (1.67-3.02)	<0.001
Citations	3.01 (2.20-4.11)	<0.001
h-index	1.93 (1.36-2.73)	<0.001
Associate professor		
Total publications	1.67 (1.00-2.79)	0.049
Citations	3.63 (2.13-6.18)	<0.001
h-index	1.93 (1.10-3.39)	0.022

## Discussion

Although recent years have seen an increase in the number of osteopathic students entering the physician workforce, their underrepresentation in general surgery is apparent [1,2]. This discrepancy is further accentuated at the academic general surgery level, where osteopathic graduates are seldom represented as faculty, as noted in our results. While the barriers to entry to university-based surgical residency programs have been well established in previous literature, very little is known about osteopathic surgeons who serve as faculty at these institutions [4,5]. Our survey of 106 university-based general surgery departments identified 70 faculty who hold a DO degree. Most of these surgeons completed a community or university-affiliated community residency program, and most completed a fellowship. We also recognized that while at the assistant professor level, MD faculty had more total publications, this discrepancy diminished at the associate professor level.

Mentors encountered during medical school, residency, and fellowship play a significant role in fostering academic advancement [9]. For some osteopathic medical students and residents who undergo their training at non-university-based programs, access to available mentors is, at times, limited [4,5,10]. This limited access to academic institutions can create challenges for osteopathic students and residents in forging relationships and obtaining letters of recommendation from nationally recognized surgical leaders. Along those lines, our study showed that there is a disparity between MD and DO surgeons holding faculty positions at university-based general surgery departments. We also demonstrated that the majority of the DO faculty were at the assistant professor rank, with only one surgeon currently serving at the professor rank.

Of the DO faculty, 64.3% completed their residency training at a community or university-affiliated residency program, and 92.9% completed a fellowship training program. This finding is consistent with reports from Boyev et al. who had previously reported that a majority of DO residents are trained primarily at community programs rather than academic centers [11]. It is likely that this lack of representation of DO physicians in academic training programs is associated with the lack of access osteopathic students have to academic surgery departments. This limitation severely affects the number of qualified mentors who can provide students with guidance, research opportunities, and endorsements for residency applications. Furthermore, it can also be hypothesized that this lack of resources may continue for those osteopathic residents at non-university-based residency programs, where funding and infrastructure for academic career advancement may be lacking [12].

Academic productivity for surgical faculty is often quantified by the total number of publications they produce during their career; this metric plays a significant role in academic career advancement. Although there are no previous studies observing the publication trends of osteopathic surgeons, previous studies have noted a discrepancy in the rate of publication between osteopathic and allopathic physicians in other fields. Merritt et al. in their study looking at the medical degree of authors in obstetrics and gynecology journals found that only 0.80% of authors held osteopathic degrees [13]. Similar trends were seen by Lammers et al. and Antony et al. in their studies analyzing the authorship trends and R01 grant funding of emergency medicine faculty, respectively [14,15]. Along those lines, our study also found that compared to their MD counterparts, DO faculty had fewer publications, citations, and lower h-index at the assistant professor level. However, we did note that this discrepancy upon promotion to associate professor level diminishes. One of the reasons this disparity is widest at the assistant professor level may be due to the differences in training that allopathic and osteopathic medical students receive. Historically, osteopathic medical school curriculums did not contain substantial research components, despite their medical students’ desire to incorporate research into their practice [12,16-18]. For this reason, osteopathic students often begin their careers with fewer publications compared to their MD counterparts [13]. However, our results suggest that DO physicians who obtain an academic general surgery faculty position are able to narrow this gap, once given adequate time to train, network, and establish themselves.

The most direct and impactful solution to the discrepancy noted at the faculty level would be to encourage osteopathic medical schools to broaden clinical and research experiences while affiliating with institutions with surgical residency programs. Since these require significant time and financial investments, it is also important to discuss ways in which change may be influenced in the short term, such as providing osteopathic students resources to nearby academic centers for mentorship and research opportunities. Furthermore, while three out of four incoming osteopathic medical students reported an interest in research, almost 50% reported that their medical school did not provide training in research methodology and literature analysis [19,20]. For this reason, osteopathic medical schools should adjust their curriculum to include research methods and analysis along with incentives for academic faculty to provide research mentorship to students. These initiatives would provide osteopathic students with a solid foundation of knowledge while also sparking an interest in academic medicine.

The primary limitation of this study is that it is observational and therefore can only suggest correlation rather than causation. Furthermore, we limited our cross-sectional survey to the top 106 university-based surgery departments. While we felt that this was an adequate representation of academic general surgery, it does not fully represent the field in its entirety. Additionally, when it came to collecting author publication data, we did not account for the type of publication and the authorship position. Lastly, due to the lack of standardization of other academic activities across surgery departments (such as leadership roles and teaching roles), we elected to use publication numbers as a surrogate for academic productivity.

## Conclusions

The discrepancy between DO and MD surgeons spans all levels of medical training, from medical students to academic faculty. Many osteopathic programs do not have direct partnerships with academic general surgery programs; thus, opportunities to find mentors and research opportunities are limited. Many osteopathic students have to go beyond their own institution to other residency programs and academic institutions to seek mentorship and research. This may have impacted both research opportunities and opportunities to network. To address this discrepancy, action must be taken by osteopathic medical schools and leaders of the field to ensure that DO students receive better access to mentorship and research support to foster an academic career.
